# Effect of Non-Woven Polyethylene Terephthalate (PET) Tissue on Fresh and Hardened Properties of Concrete

**DOI:** 10.3390/ma15248766

**Published:** 2022-12-08

**Authors:** Sifatullah Bahij, Safiullah Omary, Vincent Steiner, Françoise Feugeas, Mohammad Hashim Ibrahimkhil

**Affiliations:** 1 ICube, UMR CNRS 7357, INSA-Strasbourg, 24 Bld de la Victoire, University of Strasbourg, 67084 Strasbourg, France; 2 Department of Civil and Industrial Construction, Kabul Polytechnic University, Kabul 1010, Afghanistan; 3 Department of Building Construction Management, Kabul Polytechnic University, Kabul 1010, Afghanistan

**Keywords:** plastic wastes, non-woven tissue, fresh and physical properties, mechanical behaviors, ultrasonic pulse velocity, thermal conductivity

## Abstract

This study will investigate the effect of non-woven PET plastic tissue on the fresh, physical, mechanical, acoustic, thermal, and microstructural behaviors of concrete. Including reference specimens, non-woven fabrics were considered in two ways: (a) as a layer with four various configurations of 1-layer, 2-sides, 3-sides, and full wrapping (4-sides) to strengthen specimens, and (b) as (10 × 10) mm cut pieces with three different incorporated percentages of 0.25%, 0.50%, and 0.75%. Based on the experimental results, mechanical properties (compressive, split tensile, and flexural strengths) were remarkably improved by applying non-woven sheets as a layer. For instance, the cylindrical compressive and split tensile strengths were improved by 13.40% and 15.12% for the strengthened specimens compared to the reference specimens, respectively. Moreover, control specimens were damaged to many fragments after mechanical testing, but the samples strengthened by such fabrics or containing cut pieces were maintained and not separated into many small parts. The acoustic behavior and thermal conductivity declined by 9.83% and 19.67% with the attachment of tissue on one side and 2-sides, respectively. Acoustic behaviors decreased by 10.0%, 17.60%, and 26.30% and thermal conductivity decreased by 6.60%, 12.10%, and 15.50%, with the incorporation of 0.25%, 0.50%, and 0.75% of cut pieces, respectively. Finally, it was discovered that non-woven tissue is advised to enhance particular properties of concrete.

## 1. Introduction

In recent decades, plastic consumption has been vastly increased because of rapid urbanization and economic growth. The recycled amount is still comparatively lower and results in an increase of plastic wastes. These wastes cause many environmental problems because of the absence of enough space for landfilling and their low biodegradability [1,2,3,4,5,6]. A total of 348 million tons of plastic were produced worldwide in 2017, while 61.8 million tons of that total were produced in Europe. In 2018, this sum was enlarged to 359 million tons worldwide and to 64.4 million tons in Europe. In addition, in the same year, 32.5% of plastic wastes were recycled, 42.6% were used in energy recovery, and about a quarter (24.9%) were dumped in landfills worldwide. It is also estimated that the production of plastic will become double by 2035 and quadruple by 2050 [7,8,9,10]. Therefore, for better sustainable waste management, it is necessary to recycle and reuse plastic wastes that result in saving natural resources, decreasing pollution of the environment, and reducing embodied energy [11].

In general, plastics are classified into two groups based on their ingredients and mix proportions: (1) Thermoplastics can melt when heated and harden when cooled. Based on their usage, thermoplastics are subdivided into polyethylene terephthalate (PET), high-density polyethylene (HDPE), low-density polyethylene (LDPE), polyvinyl chloride (PVC), polypropylene (PE), and polystyrene (PS). (2) Thermosets are plastics that undergo chemical change and can be remolded or softened by heating [12,13].

Furthermore, the most used thermoplastic is polyethylene terephthalate (PET), which has a wide range of uses including blown bottles, bottles for soft drinks, containers for packing and food, etc. In 2007, a total of 10 million tons of PET plastic were used globally, which is equal to 250 billion bottles, and this amount increases nearly 15% annually. It is known that PET bottles used just once are discarded and result in plastic wastes that cause many environmental problems. Therefore, researchers have tried to discover ways to reduce or eliminate these environmental issues. The substitution of PET wastes by plastic aggregates or the addition of plastic fibers into cementitious materials [9,14] appears to be one possible way. Generally, plastic wastes can be preferably used in the substitution of aggregates in cementitious materials, enhancement of the mechanical and durability properties of roads and pavements, as insulation for building construction, raw materials for the production of textile, etc. [9]. In addition, most researchers presented that plastic wastes incorporated as aggregates or fibers have significant effects on certain properties of concrete/mortar [6,15,16,17,18,19,20].

The properties of concrete with varying percentages of coarser, flakier, and irregularly shaped plastic aggregates substituted by natural aggregates were investigated in an experimental investigation. The authors observed a substantial reduction in slump value for specimens having plastic aggregates compared to the reference samples. Additionally, the concrete matrix with waste plastic aggregates had lower drying shrinkage than normal concrete. This might be because plastic waste is impermeable and has a great capacity to absorb water while the concrete is in a fresh state [21]. Concrete workability decreased noticeably with an increase in the percentage of high-density polyethylene plastic fibers when they were added to concrete mixtures with 0%, 0.40%, 0.75%, and 1.25% [22].

Additionally, the results of an experiment on the physical and mechanical characteristics of mortars with 3%, 10%, 20%, and 50% replacement of sand with PET waste aggregates show that the compressive strength decreased as the percentage of plastic aggregates increased due to the poor bond between cement paste and plastic aggregates [18]. The research on self-compacting concrete containing 0.25%, 0.5%, 0.75%, 1.0%, 1.25%, 1.5%, 1.75%, and 2.0% of plastic fibers underlined that the compressive strength improved up to 1.5% of plastic fibers and decreased beyond this value [23]. Additionally, the flexural strength increased as the percent of PET fibers increased up to 1.5%, but it decreased for 2.0% of PET fibers [24]. Similarly, the flexural strength was improved by 10–13%, while 0.15–0.45% of 12 mm long polypropylene fibers were added into concrete mixtures [25]. On the other hand, a study was conducted on concrete mixtures using 5%, 10%, and 15% of PET aggregates in place of natural ones. The results highlighted that split tensile strength declined as the percentage of plastic aggregates increased. This can be due to the smoother surface of plastic aggregates and the presence of free water on the particle surface, which causes a weaker interaction between cement paste and plastic aggregates [26]. The study on concrete mixtures with 0%, 5%, 15%, 30%, 45%, 65%, and 85% of fine aggregates substituted by PVC trash revealed that as the amount of plastic waste increased, the tensile splitting strength of the concrete gradually declined [27].

In addition, experimental work was conducted to explore the sound velocity of self-compacting mortar containing 0%, 10%, 20%, 30%, and 50% of plastic waste as fine aggregates. The findings underline a remarkable reduction in sound velocity of specimens with plastic aggregates for every substitution. This can be seen in the cement’s hydration products, which fill the material’s existing cavities [19]. In addition, the speed of ultrasound remarkably decreased, while PET plastic aggregates were substituted by fine aggregates. This is explained by the variation in ultrasonic speed between plastic particles and concrete aggregates [28]. Finally, the authors found that the thermal conductivity of concrete decreased by 35%, 39%, 43%, and 58%, respectively, when fine aggregates were replaced with 30%, 40%, 50%, and 60% of waste plastic. Two main reasons can be identified for this: (1) the lower thermal conductivity of plastic aggregates compared to natural ones, and (2) the high porosity of concretes made with plastic aggregates [29]. Similar to this, an experiment was conducted on concrete samples with 1.0% of three different types of plastic fibers and other specimens without fibers. The outcomes indicate that thermal conductivity (*k*) significantly decreased for the specimens having PET fibers compared to the reference ones. This might be a result of plastic fibers’ lower thermal conductivity compared to cement and aggregates [30].

On the other hand, one of the most crucial topics in civil engineering is the strengthening and retrofitting of damaged or new structural elements. Concrete could deteriorate throughout the service life because of errors in design, change in usage, inadequate maintenance, material deficiencies, overloading, fire, earthquake, or other environmental aspects. Thus, researchers have considered different materials and methods for strengthening and retrofitting of structural elements [31,32,33]. In this regard, research was conducted to study the flexural behaviors of reinforced concrete (RC) beams strengthened by fiber reinforced polymer (FRP) with various configurations. The results indicate that FRP could improve the structural performances of the damaged or new structural members more effectively. Here the degree of enhancement is related to the type and number of FRPs, strengthening techniques, type of adhesive materials, etc. [34,35,36]. Additionally, an experimental study was performed to explore the flexural behaviors of RC beams strengthened with carbon fiber reinforced polymer (CFRP) sheets. The beams were strengthened at the bottom or sides using an epoxy adhesive. The results highlight that the ultimate load-carrying capacity of the strengthened beams were significantly enhanced compared to the reference ones. Such improvement was about 62–92% for the beams strengthened at the bottom, and 40–93% for the beams strengthened at sides [37].

The above bibliographic research indicates that plenty of research was performed on concrete having plastic wastes as substituted aggregates or incorporated fibers. Numerous researchers looked at concrete elements that were repaired with various materials. However, none took PET non-woven tissue into consideration as a layer to reinforce concrete samples or as cut pieces to alter specific properties of concrete. Therefore, a product of the FREUDENBERG Company named Evolon^®^ is a microfilament textile made from a combination of short and long fibers, and bounded together by chemical, mechanical, heat, or solvent treatment was used in the present study. This product offers many possibilities for tapping into new markets and developing applications. They are used for technical packaging for the automotive and electronics industry, anti-allergy encasings, or as cleaning cloths and sports towels, etc. In addition, the chosen product was made from PET, which is a type of plastic with a wide range of applications, strong mechanical properties (strength and stiffness), long durability, etc., compared to other types [38]. Additionally, this study is an extension and continuation of an earlier experimental investigation that looked only at the mechanical and transfer characteristics of concrete specimens containing non-woven fabric as a layer [39]. However, this article covers the effect of non-woven sheets on various properties of concrete specimens, either as a layer or in the form of cut pieces. In the case of non-woven sheets as a layer, the mechanical (compressive, flexural, and split tensile strengths), acoustic, and thermal properties are investigated. The fresh (workability and density), physical (dry density, porosity, and water absorption), mechanical (compressive and split tensile strengths), acoustic, and thermal properties of concrete samples are examined while non-woven sheets are cut pieces with three different percentages of 0.25%, 0.50%, and 0.75%. In contrast to the previously published article, this article contains a comprehensive analysis and discussion of the results; for instance, the relationships of strengthening configurations and percentages of cut pieces with various behaviors of concrete specimens. Moreover, the experimental data was checked by the models developed in the previous literature.

## 2. Experimental Program

### 2.1. Materials

In the current study, concrete mixtures were manufactured using locally accessible Ordinary Portland Cement known as Ghori that complied to ASTM C150 [40] with the Blaine surface area of 2900 cm^2^/gr and specific gravity of 3.041. Table 1 presents the chemical compositions of this cement.

**Figure 1:**
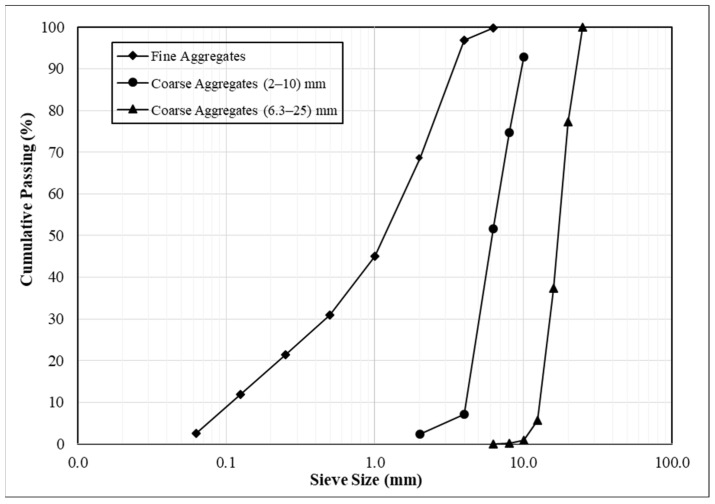
Coarse and fine aggregates’ size distributions.

In addition, two different sizes of coarse aggregates (10 mm and 25 mm) crushed from mountain rocks and fine aggregates with 6.3 mm maximum size obtained from the natural sand of a river were used in concrete mixtures. Figure 1 and Table 2 display the size distributions and other physical characteristics of both coarse and fine aggregates, respectively.

Additionally, ADIUM 150, a new generation of polycarboxylate-based water reduction agent that was confirmed by EN 934-2 [41] and obtained from the regional Isomat Company branch (Kabul, Afghanistan), was employed to enhance the workability and flowability of the concrete mixtures. The physical and chemical properties of the mentioned superplasticizer are shown in Table 3.

**Figure 2:**
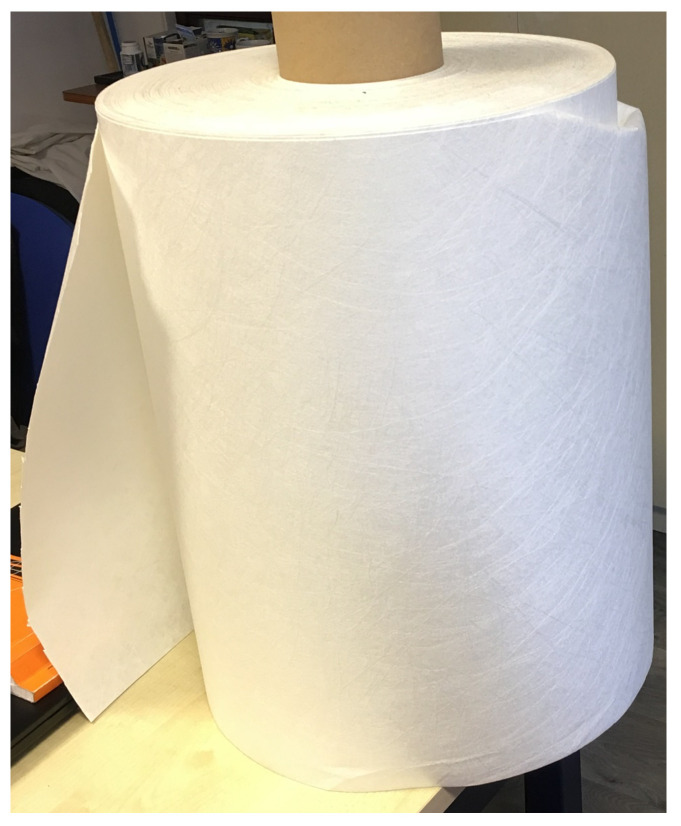
Non-woven plastic tissue (Evolon^®^ microfilaments).

Finally, non-woven plastic tissue or Evolon^®^ microfilaments were sourced from the FREUDENBERG Company (Colmar, France) [38]. These fabrics were manufactured using higher water pressure jets through splitting, entangling, and bonding. The outcome is a textile with the microfilament structure that provides mechanical strength and softness similar to woven fabrics as shown in Figure 2. These textiles are very strong and isotropic, have similar properties in all directions, and have good mechanical characteristics, which prevent them from losing their shape or uniformity. In addition, Evolon^®^ microfilaments are very durable fabrics, have very good sound absorption, and thermal insulation properties.

### 2.2. Experimental Methods

In the present study, non-woven sheets were used in concrete mixtures with two methods: (1) as a layer to strengthen samples, and (2) as (10 × 10) mm cut pieces incorporated with various percentages of 0%, 0.25%, 0.50%, and 0.75%. Based on ACI Standard Practice ACI 291.1 [42] (volume basis), the mix proportion of concrete components is calculated, which has a water-to-cement ratio of 0.45 and a total binder content of 455 kg/m^3^. The mix design with 0.0% of cut pieces was considered for the samples using non-woven sheets as a layer, while for concrete mixtures containing cut pieces of non-woven sheets, the mix proportions were slightly modified with the addition of cut pieces and superplasticizer due to lower consistency. In this case, the content of fabrics for one cubic meter is measured by taking the mass of concrete as 2133 kg. For instance, the amount of 0.50% of non-woven sheets by mass of concrete is taken as 0.50 × 2133/100 = 10.66 kg/m^3^. The detailed amount of ingredients for samples with a non-woven sheet as a layer or cut pieces are presented in Table 4.

**Figure 3:**
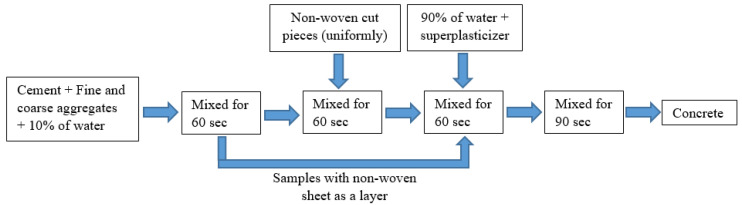
Mixing procedures of samples with non-woven sheets as a layer or cut pieces.

It is essential to consider a proper batching, mixing sequence, and mixing duration to manufacture concrete mixtures with the desired workability and homogeneity. Therefore, a tilting drum mixer was used and the mixing procedure of concrete mixtures with and without cut pieces are shown in Figure 3.

Additionally, utilizing non-woven sheets as a layer, molds were oiled for easy demolding before casting the samples, and non-woven tissues were then placed in molds in accordance with their configurations, as shown in Table 5. The concrete mixtures were then poured into the molds and vibrated using a vibrating device. After being placed in molds for 24 h, all samples were demolded, and the specimens were then allowed to cure for a maximum of 28 days at a normal temperature of (20 ± 2) °C. In addition, 100 mm cubes were prepared to measure the compressive strength, (100 × 200) mm cylinders for split tensile and compressive strengths, (100 × 70) mm cylinders for the dry density, water absorption, porosity, thermal conductivity, and ultrasonic pulse velocity and (100 × 100 × 500) mm beams to examine the flexural strength. Moreover, the specimens were tested after 7, 14, and 28 days of curing.

### 2.3. Experimental Procedures

Workability of concrete mixtures was evaluated using the ASTM C143 code requirements [43]. According to ASTM C39 standards, the compressive strength of both cube and cylindrical specimens was measured using an ADR touch SOLO 1500 compression machine with digital readout manufactured by ELE Company in Sheffield, UK [44]. In accordance with ASTM C78 standard [45], the flexural test was conducted on a beam sample. A 50 kN mini-flexural machine having a four-point loading setup with a constant shear span to the effective depth ratio of a/d = 1.25 was applied to analyze the flexural strength of beams. Moreover, the ADR touch SOLO 1500 machine, confirmed by ASTM C496, was used to explore the tensile splitting strength of cylindrical samples [46]. Additionally, the physical properties (dry density, porosity, and water absorption) of concrete were measured based on ASTM C642-13 [47] code considerations. The surface probe of the ISOMET 2104 equipment manufactured by Applied Precision in Bratislava, Slovakia was used to observe the thermal conductivity of concrete [48]. Finally, the ultrasonic pulse velocity test was carried out using ELE Pundit Plus equipment manufactured by ELE Company in Sheffield, UK with a direct method on 100 mm cubes in accordance with ASTM C597 standards [49]. The pulse velocity values were computed by dividing the cube dimension by the transit time. Each concrete property was evaluated based on 3 samples, and the errors were calculated using the standard deviation and displayed in each graph. In addition, the data were analyzed based on the percentages of improvement and reduction to examine the significance criteria for each property of concrete as shown in Table 6.

## 3. Results and Discussions

### 3.1. Non-Woven Sheets as a Layer to Strengthen Specimens

#### 3.1.1. Fresh Properties

The same mix design was employed for both the reference samples and the samples with non-woven tissue because such sheets were used as a layer with various configurations on the specimens’ exterior faces. Because of this reason, there was no effect of non-woven sheets on the fresh properties of such concrete. However, the slump test was performed on three samples to determine the workability of concrete mixtures made based on the mix design. The average slump value was 158 mm, which is in the standard range of (150–180) mm. The fresh density was obtained from three samples and their average was 2680.4 kg/m^3^.

#### 3.1.2. Mechanical Properties

(a) Compressive strength

**Figure 4:**
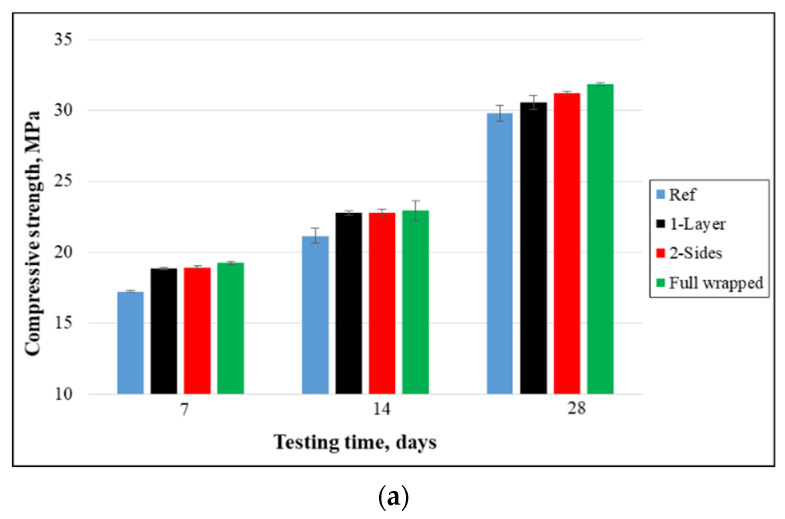

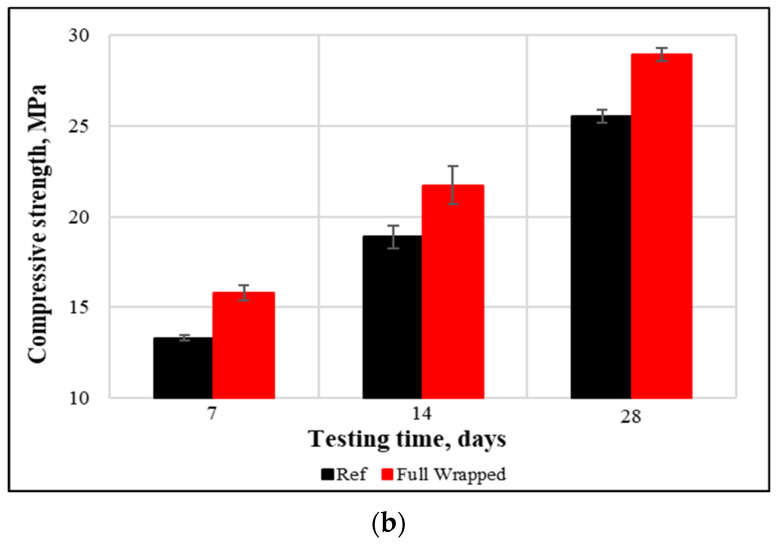
Compressive strength: (**a**) cubes and (**b**) cylinders.

Figure 4 displays the compressive strength findings and comparisons at 7, 14, and 28 days of curing. The outcomes indicate that the application of non-woven tissue as a layer has a negligible effect on the compressive strength of cubical specimens, which means that the cubical compressive strength has slightly increased when specimens were strengthened with the different configurations of tissue. However, the compressive strength of cylindrical samples strengthened by such sheets was enhanced compared to the reference samples as shown in Figure 4b. After 28 days of curing, the compressive strength of cubical samples strengthened as 1-layer, 2-sides, and 4-sides increased negligibly by 2.5%, 4.7%, and 6.8%, respectively, in comparison to the reference samples. The cylindrical compressive strength of strengthened samples increased significantly by 13.4% over control specimens during the same curing period, which is because such tissue has the capacity to constrain crack extension, alter the cracking path, and slow the pace at which cracks grow. Additionally, more energy was needed for the final collapse and the spread of cracks within samples.

**Figure 5:**
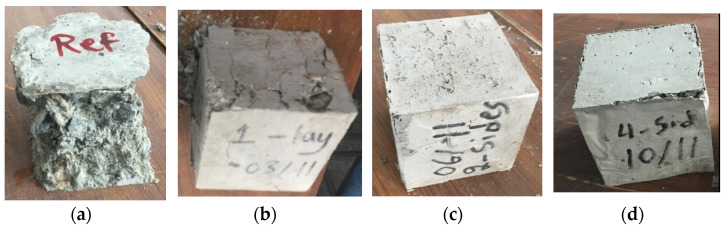
Cracking mechanism of cubes. (**a**) Reference, (**b**) 1-layer, (**c**) 2-sides, and (**d**) 4-sides.

**Figure 6:**
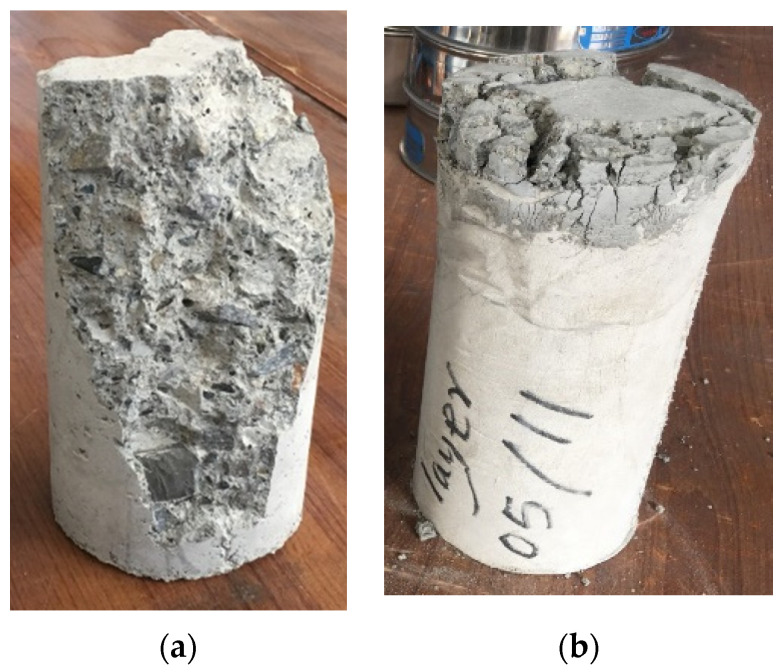
Cracking mechanism of cylinders. (**a**) Reference and (**b**) covered samples.

As indicated by the fracture pattern, the reference specimens clearly show numerous cracks after the ultimate load, whereas the specimens with the 1-layer, 2-sides, and 4-sides strengthening configurations did not exhibit many cracks. It indicates that samples were maintained by tissue, and particularly the strengthened sides as shown in Figure 5. Moreover, the cubes strengthened as full wrapped (4-Sides) were completely confined by non-woven sheets with only a few detachments at the surface, where the load was applied as shown in Figure 5d. Additionally, non-woven tissue play a heavier role in the case of cylindrical samples to prevent the specimens from being separated, as seen in Figure 6. This performance of tissue may be helpful during abrupt failure and particularly in earthquake damage. In the event of an earthquake, structural elements strengthened by such tissue will be safer than members without non-woven fabrics.

Even though the research study did not consider plastic non-woven tissue for strengthening purposes, the improvement of the compressive strength was verified by the previous experimental works, whereas concrete specimens were strengthened by fiber reinforced polymer (FRP) sheets using various methods. However, there is a difference between the outcomes of the current study and the previous studies due to great difference between material strengths, adhesive materials, etc. [50,51,52].

(b) Flexural strength

**Figure 7:**
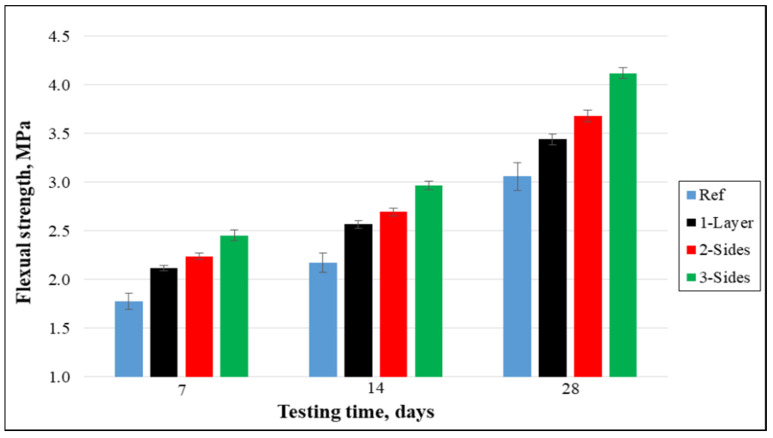
Flexural strength of concrete samples.

The flexural strength was found under a four-point loading setup. The outcomes clearly demonstrate that the flexural strength was enhanced significantly or highly significant for the strengthened beams compared to the reference beams Figure 7 demonstrates that after 28 days of curing, the flexural strength enhanced by 12.42%, 20.26%, and 34.64% for the beams strengthened at 1-side, 2-sides, and 3-sides, respectively, compared to the control beams. This is due to the attachment and strengthening of the sheet on concrete beams that leads to postponing the crack propagation, and finally, more load is needed for beam damage. The ultimate load capacity of 3-sides strengthened beams was highest, followed by 2-sides and 1-layer.

**Figure 8:**
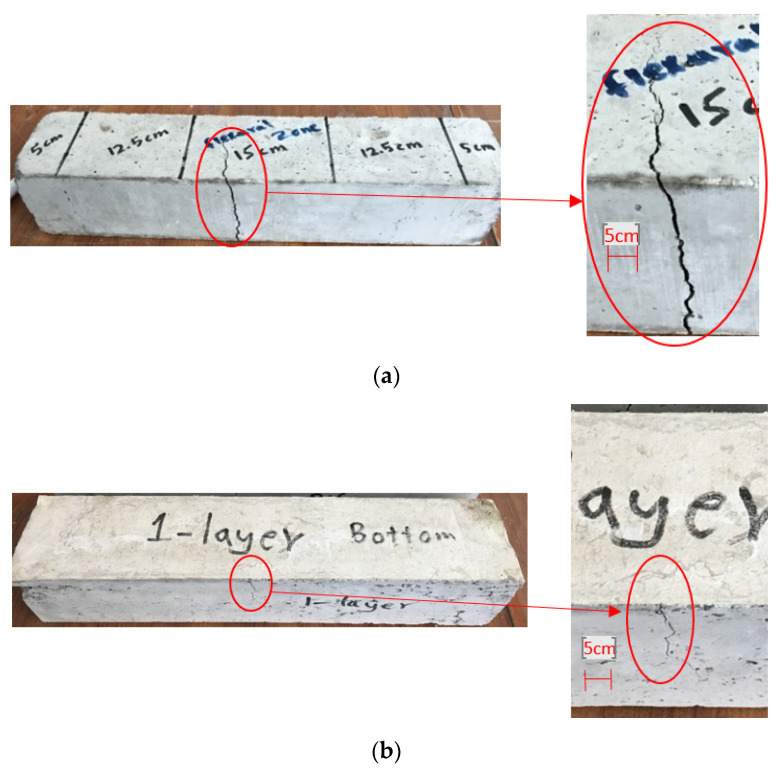

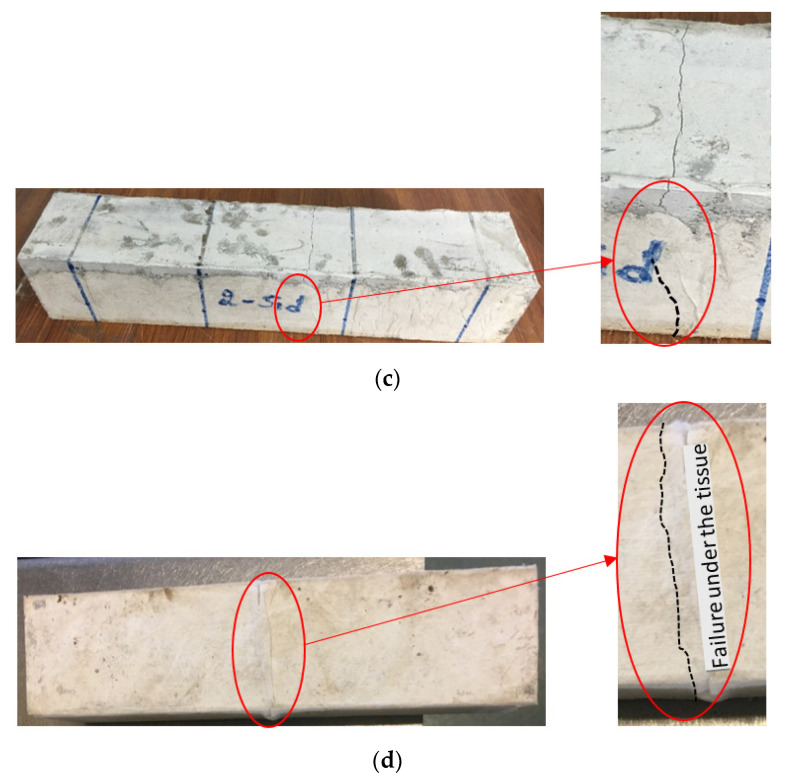
Cracking pattern of beams. (**a**) Reference, (**b**) 1-layer, (**c**) 2-sides, and (**d**) 3-sides.

Additionally, the cracking mechanisms of all reinforced beams were similar to those of the control beams, where flexural cracks started at the middle of the beams between two loading points, spread to the depth of the beams, and were then followed by a single, widened crack until the ultimate failure. However, the final modes of failure for reference and strengthened beams were observed as follow:

- *Reference beam:* The reference beams failed abruptly and completely divided into two parts after failure and had wider ultimate cracks during failure as shown in Figure 8a.
- *1-layer:* After the ultimate loading, beams containing a single layer of non-woven tissue at the bottom were damaged but not divided into two portions and were maintained by the layers of tissue. The width of crack was remarkably decreased as was observed for the reference beams. In addition, the layer of non-woven sheets was locally detached at one or both sides of the crack but not broken as shown in Figure 8b.
- *2-sides:* These beams were broken at the bottom of the flexural zone. The cracks were seen at the bottom and top of the beams but were covered completely by non-woven sheets at vertical faces. In addition, the tissue was slightly broken at the bottom of beams but remained unbroken close to the top of beams as shown in Figure 8c.
- *3-sides:* The failure occurred at the bottom of the beams under non-woven sheets. In this case, the non-woven tissue was not broken but was detached around the entire cross-section and such detachment was more significant at the bottom of the beams. Moreover, the samples were entirely maintained by the tissue and were not separated into two parts as shown in Figure 8d.

There is no bibliographic research that considered such fabrics to retrofit concrete samples. However, many researchers conducted similar research but considered different materials such as FRP, cementitious materials, etc., to strengthen concrete beams. However, the current outcomes match the results of previous research, and they also found a remarkable improvement with the use of strengthening materials. In addition, this enhancement was more significant for U-wrapping, and 2-sides compared to the 1-sides. The difference between the current results and previous results is only linked to the properties of strengthening materials [53,54,55,56].

(c) Split tensile strength

**Figure 9:**
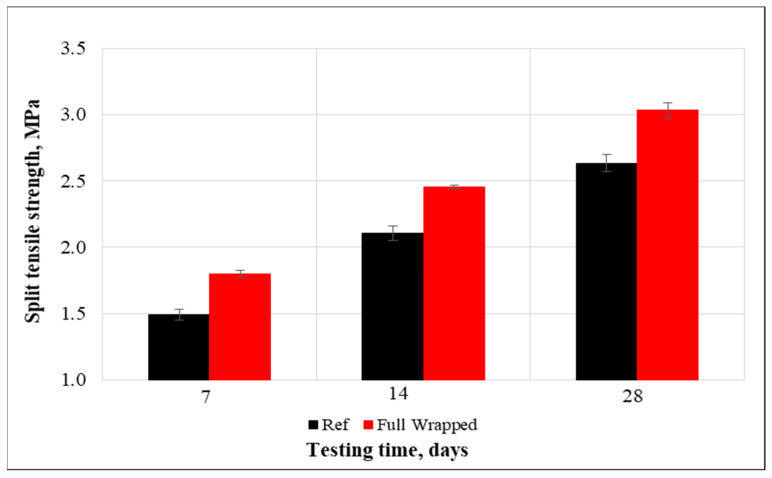
Tensile split strength of concrete.

The split tensile strength test uses a cylinder that splits across the vertical diameter to indirectly assess the tensile strength of concrete specimens. The outcomes of cylinders tested under the tensile splitting setup are presented in Figure 9. The results clearly illustrate that the tensile splitting strength after 7, 14, and 28 days of curing have enhanced significantly for cylinders covered with tissue compared to the non-covered samples. After 28 days, the control specimens had a tensile split strength of 2.63 MPa, and this strength was increased by 15.12% to 3.03 MPa for the reinforced specimens, which is explained by the non-woven fabrics’ ability to bridge cracks and prevent samples from early damage and necessitating more effort to fail the specimens.

**Figure 10:**
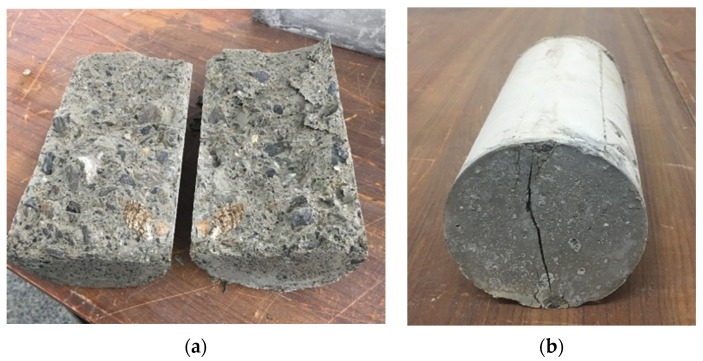
Failure mechanism of samples after split tensile strength. (**a**) Reference and (**b**) covered samples.

In addition, the control specimens failed with the brittle fracture and were separated into two entirely parted fragments. Contrarily, cylinders wrapped with the tissue were not separated but were kept together. This indicates that samples could withstand larger splitting loads when plastic tissues are attached, and the ultimate failure will be without separation as seen in Figure 10. These outcomes highlight the ability to prevent catastrophic failure in case of excessive loads on structural elements.

#### 3.1.3. Transfer Properties

(a) Ultrasonic pulse velocity (UPV)

This test measures the speed of the ultrasonic pulses’ transit time. The electro-acoustical transducer used in the UPV test produces ultrasonic pulses with a frequency of 50–58 kHz that travel from one surface of the element to the other. The transit time of ultrasonic pulses is influenced by the density, elastic and other properties of the material being tested.

**Figure 11:**
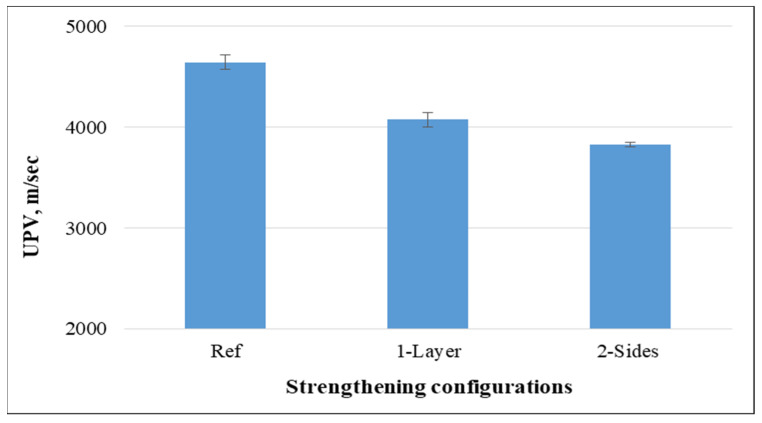
Ultrasonic pulse velocity of concrete.

Figure 11 shows the outcomes of the UPV test. The results indicate that the attachment of non-woven sheets on concrete samples led to a significant reduction of the UPV value compared to reference samples. Compared to the control specimens, the UPV value for the specimens with non-woven tissue on one or two sides fell by 12.25% and 17.63%, respectively, after 28 days of curing. This is explained by the fact that the plastic’s pulse velocity is lower than that of concrete. Additionally, it may be related to the high porosity of non-woven tissue.

(b) Thermal conductivity

In order to understand how much heat is transferred through conduction, one of the most important characteristics is the concrete’s thermal conductivity (*λ*). Building energy consumption is directly impacted by the quantity of heat loss via the walls and roof. The amount of heat transfer depends on the physical and mechanical properties of concrete materials, mix proportion of concrete, moisture content, etc.

**Figure 12:**
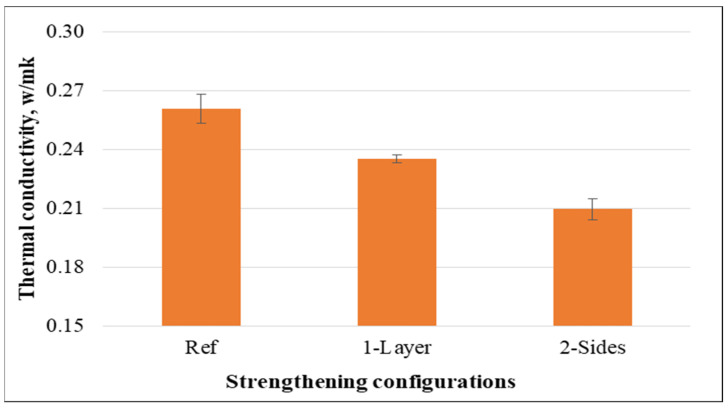
Thermal conductivity of concrete specimens.

It was observed that the amount of heat transmission was significantly reduced when non-woven tissues were attached to concrete specimens as opposed to the control specimens. Figure 12 shows that after 28 days of curing, the thermal conductivity of the specimens with non-woven tissue on one or both sides fell by 9.83% and 19.67%, respectively, compared to the control specimens. This is explained by the fact that plastic exhibits lower heat transmission than concrete mixtures. Therefore, the usage of non-woven fabrics as a layer could improve the thermal and acoustic properties of cementitious materials.

**Figure 13:**
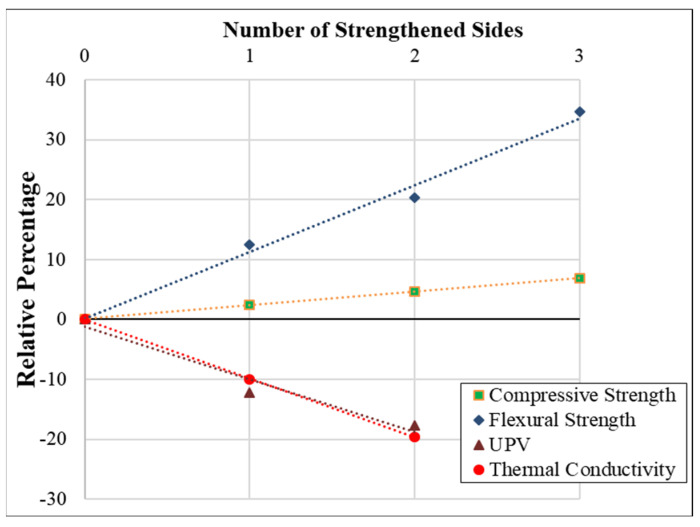
Relationship between various concrete characteristics and strengthening setups.

In addition, the correlations between strengthening configurations and concrete behaviors, such as cubical compressive strength, flexural strength, thermal conductivity, and UPV, are plotted and displayed in Figure 13. The number of strengthened faces and concrete properties are well correlated according to the R-squared correlation coefficient.

### 3.2. Non-Woven Sheets as Cut Pieces Incorporated in Concrete Mixtures

#### 3.2.1. Fresh Properties

(a) Workability

**Figure 14:**
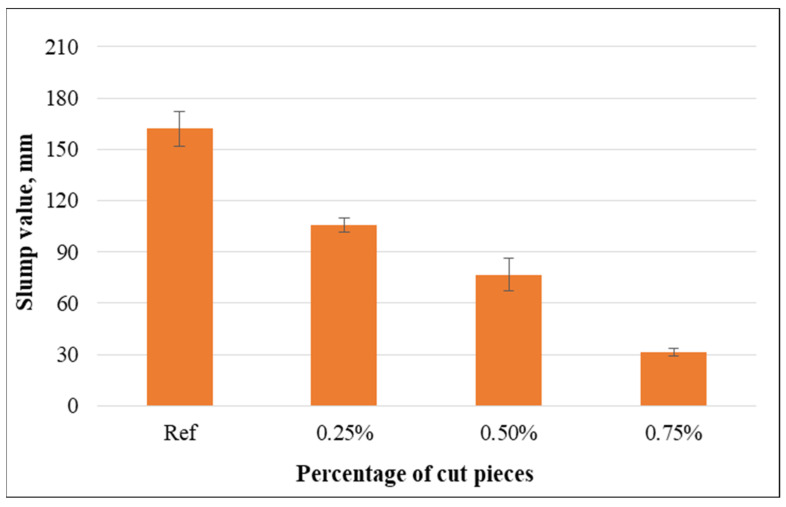
Relationship between slump values and volume fraction of non-woven tissue.

The slump values of concrete mixtures with various percentages of cut pieces are shown in Figure 14. The addition of cut pieces very significantly alters the workability properties of concrete mixtures. This indicates that the workability of mixtures containing such fabrics was lower than for the control samples, and it became even lower as the volume fraction of non-woven fabrics increased. Specifically, concrete mixtures showed slump reduction by 34.8%, 52.7%, and 80.7%, while the percentage of cut pieces varied from 0.25% to 0.75%. This is attributed to the fact that the incorporation of cut pieces modifies the viscosity of the mix due to the absorption of a large amount of water. Additionally, non-woven tissue obstructs the flow of cement paste because of the enormous surface area and ability to adhere to both fine and coarse aggregates, forming a mesh-like structure.

The reduction of slump value with the incorporation of cut pieces was confirmed by previous research. The workability decreased when plastic fibers were added, and this reduction was more pronounced at higher percentages [20,57]. This is caused by the fibers, which stick to the mixtures’ fine and coarse particles and produce a mesh-like structure.

(b) Fresh density

The fresh density of concrete illustrates its mass per unit volume at the fresh state. The fresh density depends on the mix proportion, characteristic of concrete constituents, etc.

**Figure 15:**
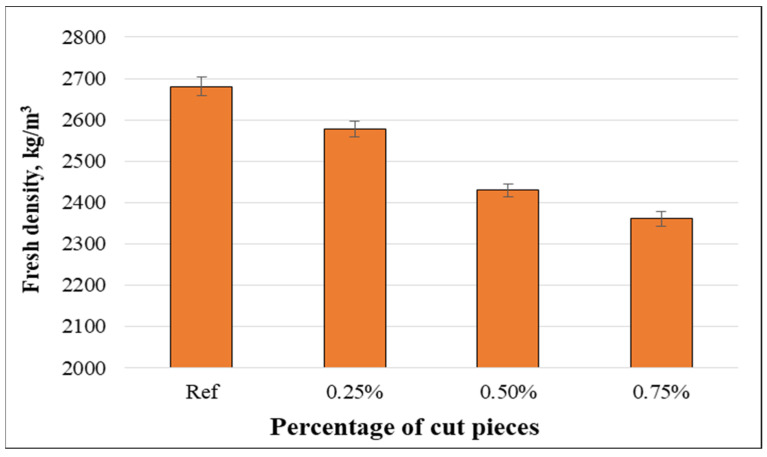
Relationship between fresh density and volume fraction of non-woven tissue.

Wet densities vary from 2680.4 kg/m^3^ to 2360.6 kg/m^3^ as presented in Figure 15. The wet density decreased as cut pieces of non-woven fabrics were incorporated into concrete mixtures. It is observed that the concrete mixtures without fabrics had the highest wet density, but concrete containing 0.75% of such fabrics had the lowest density. This can be attributed to the fact that plastic fabrics had a lower specific gravity than cement and aggregates, and plastic tissue needs more water to improve the density.

Even though prior experimental studies incorporated other kinds of plastic materials with various properties into concrete mixtures, similar trends were observed as in the present study. The outputs indicate that the fresh density has decreased with the addition of plastic fibers and decreased even more for higher percentages. This might be because plastic fibers have a lower specific gravity (1.12) than concrete components [23].

#### 3.2.2. Physical Properties

(a) Dry density

**Figure 16:**
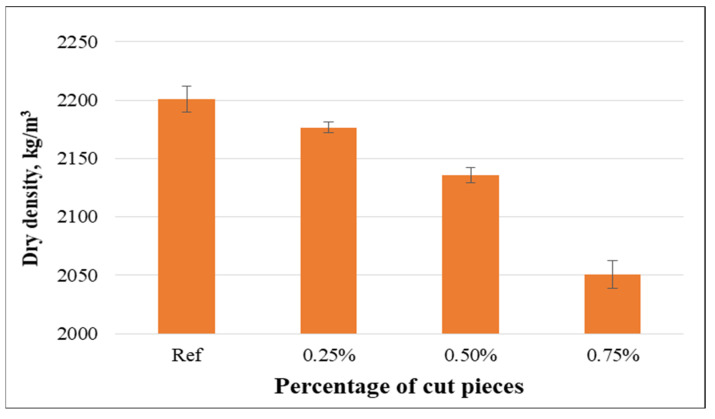
Relationship between dry density and volume fraction of non-woven tissue.

The bulk densities of studied concretes were within the range of 2201 kg/m^3^ to 2084.9 kg/m^3^. The use of non-woven tissue resulted in an insignificant reduction of dry density because non-woven fabrics were lighter than cement and aggregates, and incorporation of such fabrics resulted in porous concrete compared to the control. Distinct changes in the dry density were detected for a higher volume fraction of tissue, as presented in Figure 16.

(b) Porosity and water absorption

**Figure 17:**
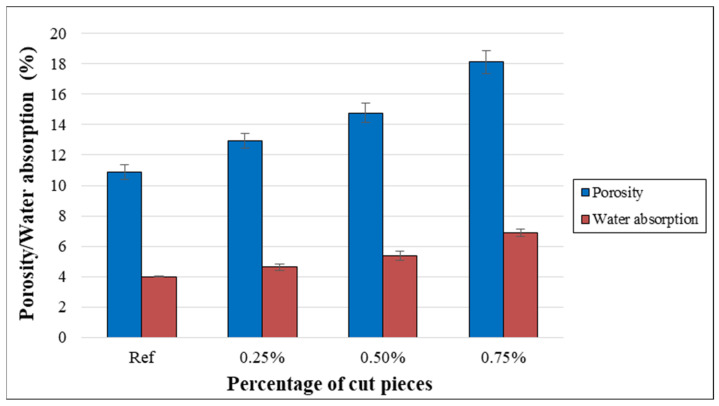
Relationship between porosity/water absorption and volume fraction of non-woven tissue.

The porosity of concrete specimens with different percentages of non-woven cut pieces is shown in Figure 17. The outcomes illustrate that the porosity has increased significantly or very significantly when cut pieces were incorporated into concrete matrixes. In addition, such an increase was more significant for concrete having a higher percentage of fabrics, which is due to the weak transition zone, macro cracks, and voids present in the interfacial transition zone between the plastic cut pieces and the cement paste.

In addition, the water absorption was calculated for concrete specimens containing non-woven plastic tissue from 0% to 0.75%. The outcomes clearly demonstrate that concrete samples with plastic fabrics had significantly or very significantly higher water absorption than the reference samples. This increase was more remarkable for higher volume fractions of plastic fabrics as shown in Figure 17. This can be attributed to the fact that non-woven fabrics absorbed most of the water and the incorporation of such fabrics resulted in porous concrete compared to control specimens.

Although no research was available in the literature that considered cut pieces of non-woven sheets incorporated into concrete mixtures, the effect of non-woven tissue on the physical properties was confirmed by previous investigations. These found that the dry density decreased, and water absorption and porosity increased with the incorporation of plastic fibers and these variations were more remarkable for higher percentages [25,58].

#### 3.2.3. Mechanical Properties

(a) Compressive strength

**Figure 18:**
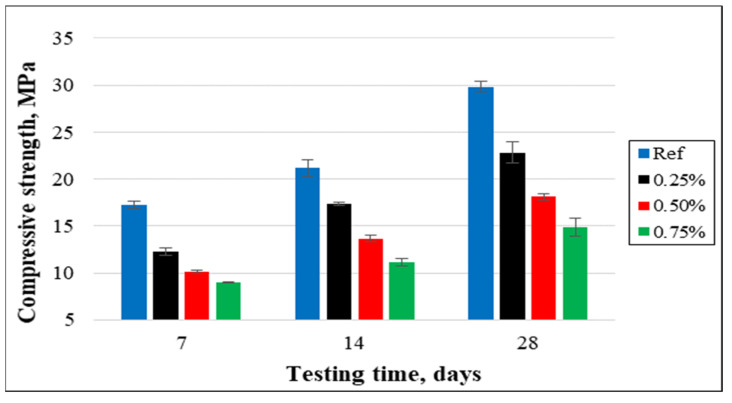
Cube compressive strength of concrete specimens containing cut pieces.

**Figure 19:**
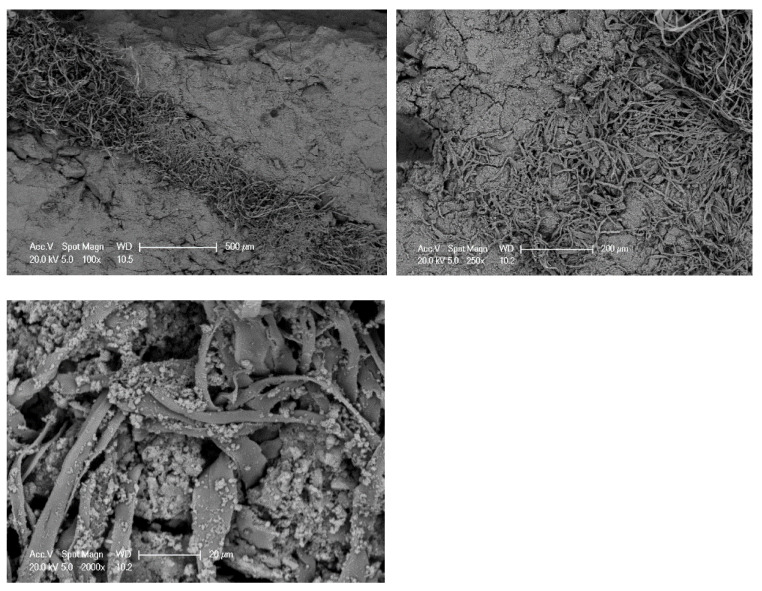
ITZ between cement paste and cut pieces of non-woven sheets.

The compressive strength of concrete specimens with different percentages of cut pieces is in Figure 18. The outcomes noticeably reveal that the compressive strength decreased significantly or very significantly with the increase of cut pieces in the concrete mixtures. After 28 days of curing, the compressive strength of the reference samples was 29.81 MPa and such strength decreased to 22.82 MPa, 18.10 MPa, and 14.84 MPa for samples having 0.25%, 0.50%, and 0.75% of non-woven fabrics, respectively. This is attributed to the weak interfacial transaction zone (ITZ) between cement paste and cut pieces as shown in Figure 19.

**Figure 20:**
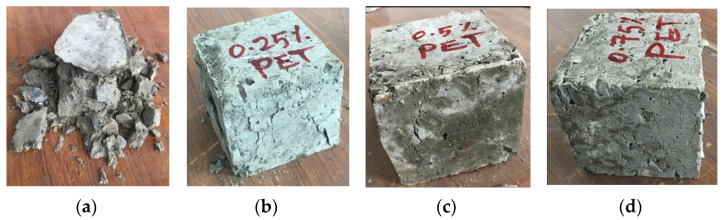
Cracking mechanisms after the compression test. (**a**) Reference, (**b**) 0.25%, (**c**) 0.50%, and (**d**) 0.75%.

The cracking pattern made it obvious that samples without cut pieces were broken into numerous pieces after the maximum force. However, specimens with 0.25%, 0.50%, and 0.75% were not separated into many parts and were maintained by such small pieces, especially for the cubes containing 0.75% of cut pieces. This is attributed to cut pieces that act as fibers inside the concrete samples and prevent the pieces of concrete from separation as shown in Figure 20.

(b) Split tensile strength

**Figure 21:**
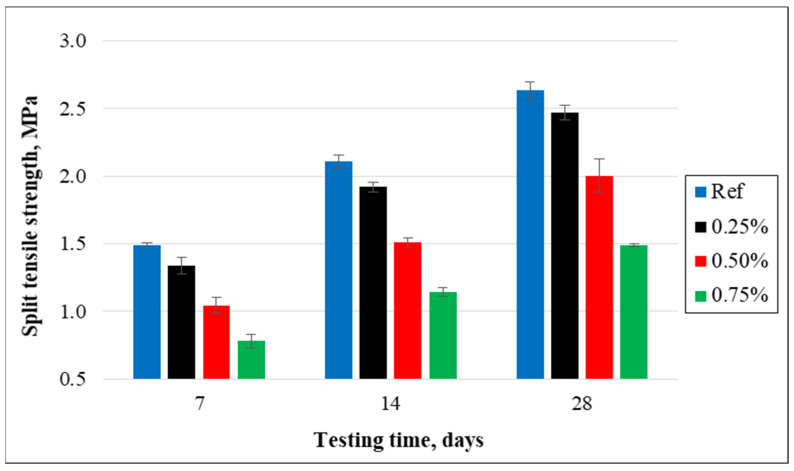
Split tensile strength of concrete specimens containing cut pieces.

Figure 21 shows the split tensile strength of concrete samples having different amounts of cut pieces and control samples. The findings noticeably reveal that the split tensile strength was reduced insignificantly for a lower percentage of cut pieces but decreased very significantly for higher percentages of cut pieces in concrete mixtures. This is attributed to the weak bond and adhesive properties between cement paste and cut pieces of non-woven fabrics. After 28 days of curing, the tensile split strength decreased by 6.6%, 31.4%, and 76.9% for the concrete samples having 0.25%, 0.50%, and 0.75% of cut pieces, respectively, compared to the control specimens. It can be highlighted that the use of non-woven sheets as cut pieces presents a negative effect on mechanical properties.

**Figure 22:**
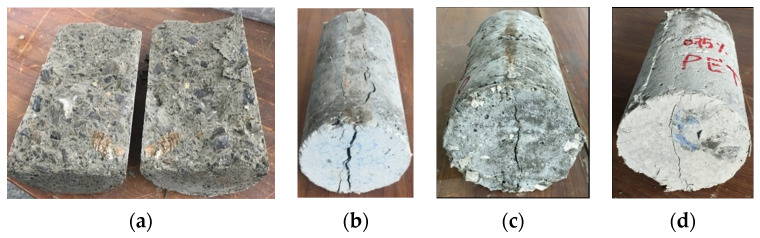
Cracking mechanisms after the split tensile test. (**a**) Reference, (**b**) 0.25%, (**c**) 0.5%, and (**d**) 0.75%.

Even though small pieces of non-woven fabrics were not beneficial for split tensile strength, it was clearly observed from the cracking pattern that samples without cut pieces were divided into two separate parts, while samples with 0.25%, 0.50%, and 0.75% were not parted into two parts and were in such small pieces from splitting. In addition, it can clearly be seen in the pictures that the width of the splitting crack was reduced considerably with the increase of percentage of cut pieces as shown in Figure 22.

In addition, the current results do not comply with the literature that present an improvement in the mechanical strengths after the introduction of plastic fibers. This is attributed to the mechanical properties of the materials themselves [17,20,30].

#### 3.2.4. Transfer Properties

(a) Ultrasonic pulse velocity (UPV)

**Figure 23:**
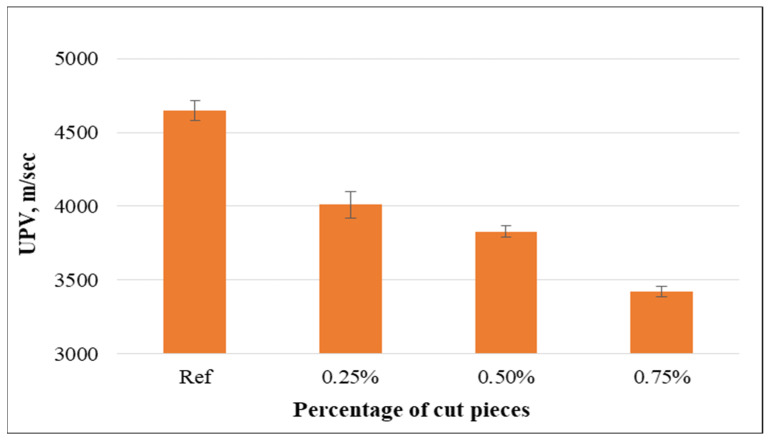
Relationship between UPV and volume fraction of non-woven cut pieces.

The UPV value was measured for concrete mixtures without fabrics and with 0.25%, 0.50%, and 0.75% of cut pieces. The results in Figure 23 highlight that the UPV value decreased significantly when plastic fabrics were added into concrete mixtures and such a decrease was more significant for higher percentages. Furthermore, the reductions for the specimens containing non-woven tissue as 1-layer and 2-sides were 12.3% and 17.6%, while these reductions were 10.0%, 17.6%, and 26.3% for the specimens having 0.25%, 0.50%, and 0.75% of cut pieces, respectively, compared to the control specimens, which means that applying non-woven sheets as a layer or cut pieces is beneficial to improve the acoustic properties of concrete.

(b) Thermal conductivity

**Figure 24:**
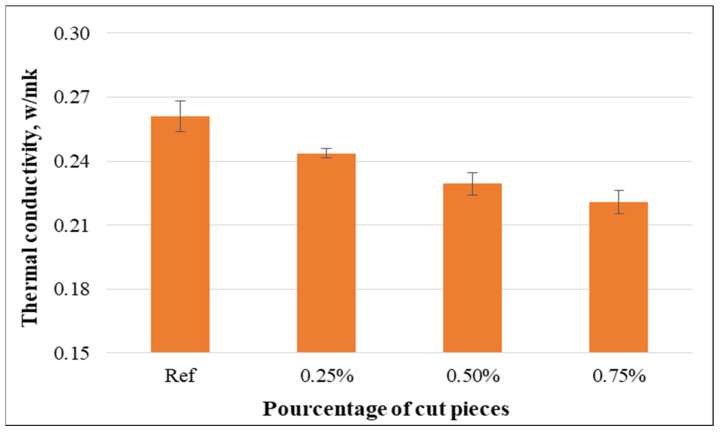
Thermal conductivity of concrete specimens containing cut pieces.

Compared to the control specimens, the thermal conductivity of the specimens containing cut pieces decreased. Furthermore, the reduction was more significant for the samples containing higher percentages of cut pieces as shown in Figure 24. This can be explained by the fact that non-woven fabrics have a lower thermal conductivity and that concretes containing non-woven fabrics tend to have a higher porosity. Moreover, thermal conductivity decreased by 9.8% and 19.7% for the samples having non-woven tissue as 1-layer and 2-sides, while such property decreased by 6.6%, 12.1%, and 15.5% for the specimens having 0.25%, 0.50%, and 0.75% of cut pieces, respectively, compared to the reference specimens. Therefore, non-woven sheets as a layer or cut pieces could be preferably used to improve thermal insulation of concrete specimens.

Even though the effect of cut pieces incorporated into concrete mixtures on thermal behaviors of concrete was not previously studied, the data of the current study regarding the transfer properties are in good agreement with previous research that considered other types of plastic wastes. This means that previous authors also found a decrease in transfer properties for plastic fiber-reinforced concrete [59,60].

## 4. Discussion

**Figure 25:**
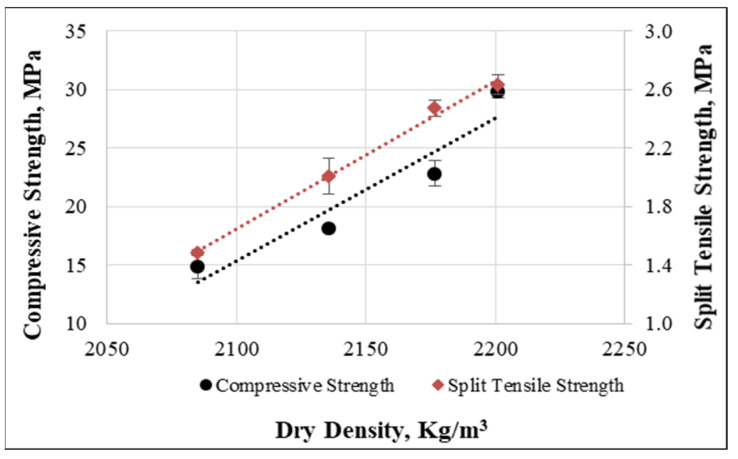
Relationship between dry density, compressive and tensile split strengths.

From the experiments, the relationship between dry density and compressive and split tensile strengths was plotted for concrete mixtures with 0, 0.25%, 0.50%, and 0.75% percentages of cut pieces after 28 days of curing. Figure 25 shows the best fit line (R^2^ = 0.99) for the split tensile strength obtained from specimens having various dry densities. However, for compressive strength, a slight variation was observed while density was changed, but the relation between dry density and compressive strength still had the best fit line (0.90), as shown in Figure 25. Finally, the relationship was plotted in two equations to predict the compressive and tensile split strengths from the dry density of concrete:

$$\begin{array}{l} f_{c}=0.1214\rho_{d}-239.61\\ f_{t}=0.0101\rho_{d}-19.567 \end{array}$$

In the above equations, $f_{c}$ is cube compressive strength, $f_{t}$ is tensile split strength, and $\rho_{d}$ is the dry density of concrete.

**Figure 26:**
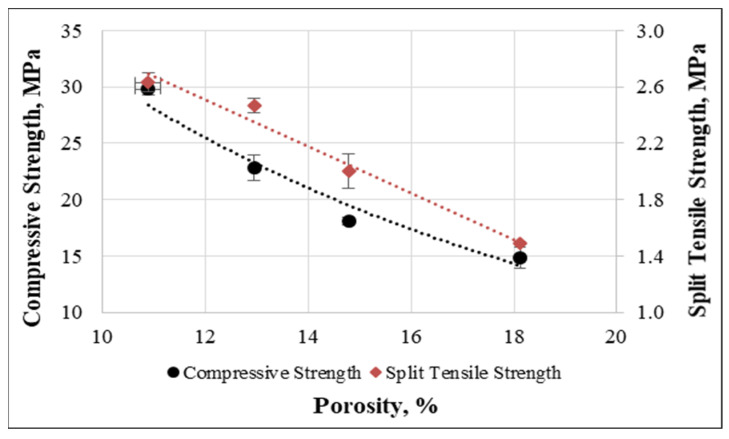
Relationship between porosity, compressive and split tensile strengths.

The relationship between the porosity and compressive and split tensile strengths are shown in Figure 26. The trends clearly indicate that R^2^ is equal to 0.96 and 0.97 for the compressive strength and split tensile strengths, respectively. Finally, from the above correlations, the following equations were obtained to estimate the compressive and split tensile strengths in terms of the porosity of concrete:

$$\begin{array}{l} f_{cm}=80.589e^{-0.096P}+50.107\\ f_{ctm-sp}=-0.1655P+4.4954 \end{array}$$

In the above equations, $f_{cm}$ is cube compressive strength, $f_{ctm-sp}$ is split tensile strength, and P is porosity of the concrete.

**Figure 27:**
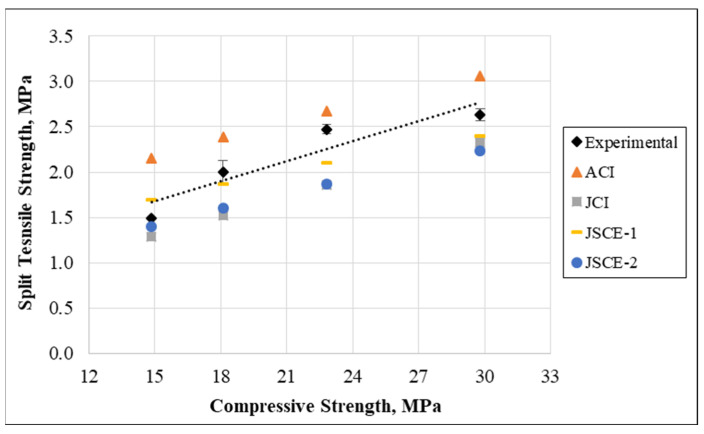
Relationship between compressive and tensile split strengths.

Furthermore, the splitting tensile strength was plotted versus the compressive strength from the experiment and to those developed by past investigators who worked on the incorporation of waste plastics in concrete mixtures. It is clearly observed from Figure 27 that there is a slight safety related to the relationship obtained from the ACI 318-11 [61], while the JCI [62], JSCE-1 [63], and JSCE-2 [64] are quite conservative when applied to the current research data. However, overall, the proposed model has a remarkable accuracy based on the present research data and previous models.

**Figure 28:**
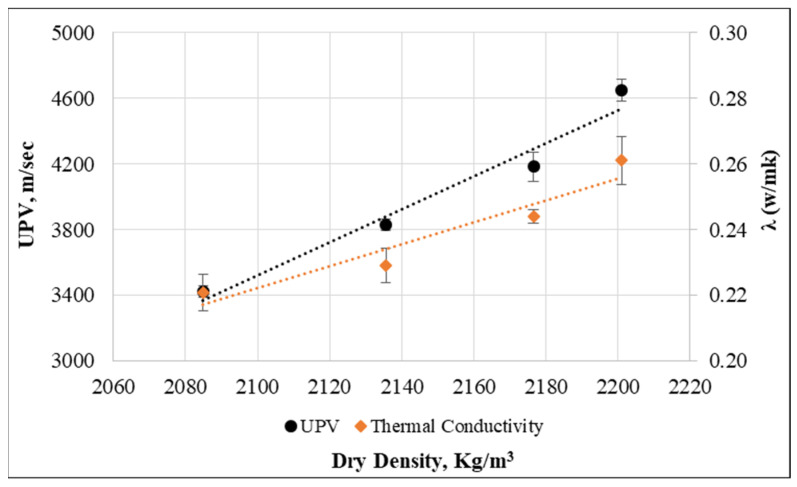
Relationship between UPV, thermal conductivity, and dry density.

Moreover, the UPV and thermal conductivity are greatly dependent on the dry density of concrete. Therefore, the relationship of dry density with UPV and thermal conductivity was plotted to predict such values while only the dry density of concrete is measured. Figure 28 presents these relationships, and it can be clearly observed that UPV and thermal conductivity has a best-fitted line with dry density. The R^2^ values are 0.96 and 0.92 for UPV and thermal conductivity, respectively. Here, the UPV value and thermal conductivity can be found from dry density with the help of the following formulas:

$$\begin{array}{l} V=17.451e^{0.0025\rho_{d}}\\ \lambda =0.0118e^{0.0014\rho_{d}} \end{array}$$

In the above equations, V is UPV value, λ is thermal conductivity, and $\rho_{d}$ is the dry density of the concrete.

**Figure 29:**
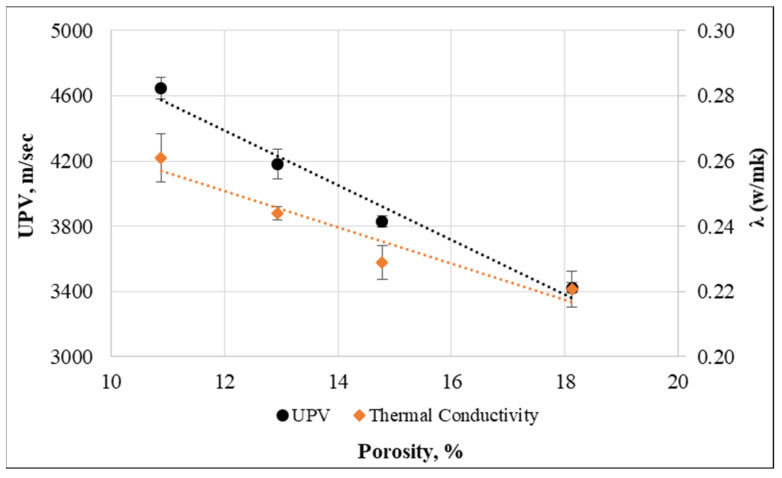
Relationship between UPV, thermal conductivity, and porosity.

Additionally, Figure 29 illustrates the correlation between concrete’s UPV, thermal conductivity, and porosity. Thermal conductivity and UPV with the dry density have R-squared correlation coefficient values of 0.92 and 0.97, respectively, indicating a strong relationship between the variables.

**Figure 30:**
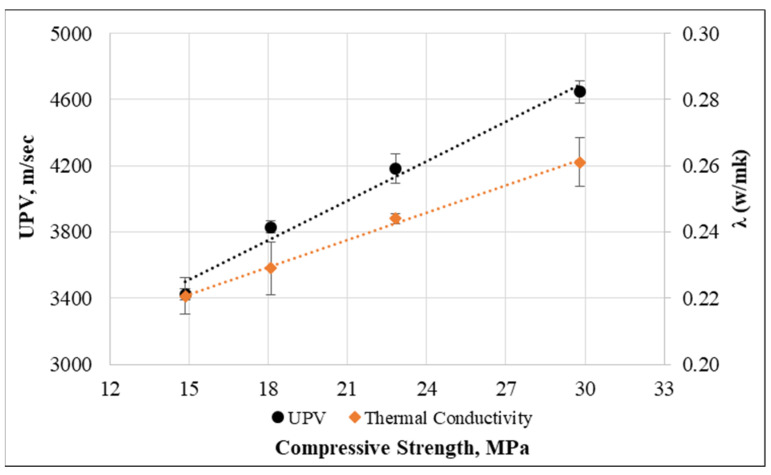
Relationship between UPV, thermal conductivity, and compressive strength.

Furthermore, Figure 30 displays the relationship between UPV, thermal conductivity, and the compressive strength of concrete. The lines trend and their R^2^ coefficient clearly indicate that UPV and thermal conductivity could correlate with the compressive strength in an accurate manner. Here, the UPV value and thermal conductivity can be found from the compressive strength with the help of the following formulas:

$$\begin{array}{l} V=79.359f_{cm}+2323.1\\ \lambda =0.0027f_{cm}+0.1803 \end{array}$$

In the above equations, V is UPV value, λ is thermal conductivity, and $f_{cm}$ is the compressive strength of the concrete.

**Figure 31:**
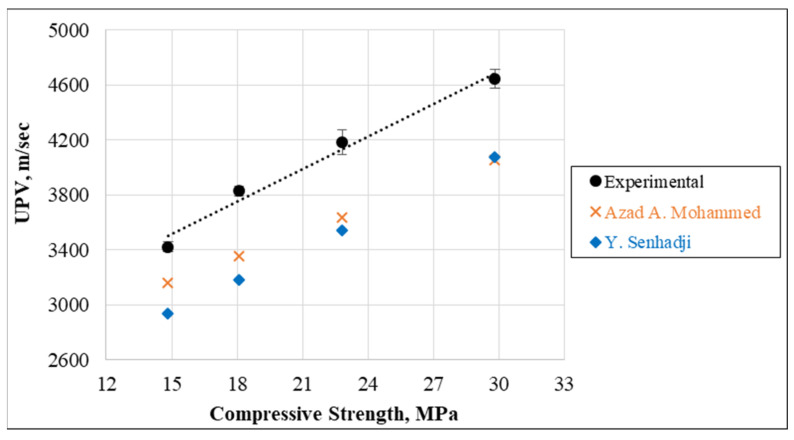
Relationship between UPV and compressive strengths [27,65].

Additionally, Figure 31 plots and displays the correlation between UPV and the compressive strength. It can be clearly observed from the R^2^ correlation coefficient (0.98) that for reference concrete mixtures and mixtures containing 0.25%, 0.50%, and 0.75% of cut pieces, the relationship between UPV and compressive strength is very good. Thereafter, the present data were predicted by models developed by past investigators who worked on the incorporation of waste plastics in concrete mixtures. Even though the present model has a slight safety related to the relationship obtained from [27,65] but still has good accuracy in terms of the present data and is validated by previous experiments.

**Figure 32:**
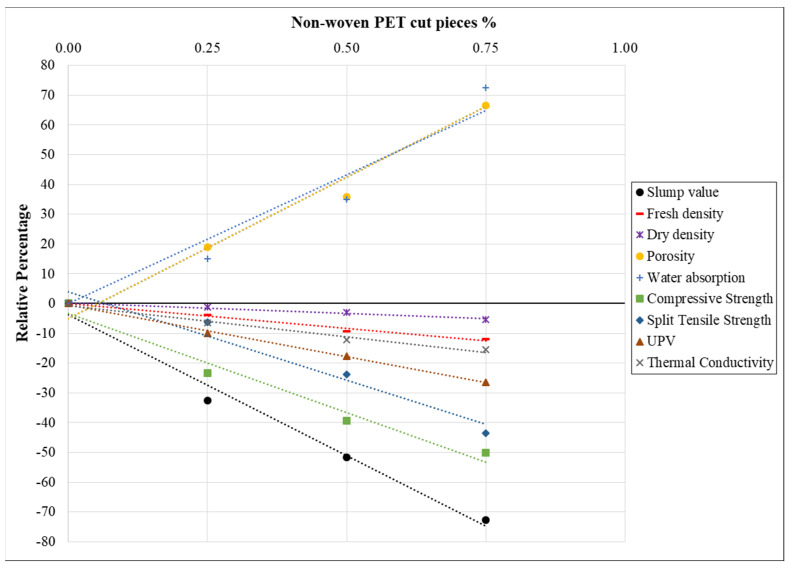
Relationship between percentages of non-woven cut pieces and various properties of concrete.

Finally, Figure 32 illustrates the correlations between the percentages of cut pieces and several concrete properties, including fresh, physical, mechanical, thermal, and acoustic. The R^2^ correlation coefficients clearly indicate that the percentages of cut pieces have an excellent correlation with certain properties of concrete.

## 5. Conclusions

This study highlights the possibility of using plastic non-woven tissue in concrete. The key findings are outlined below:

- Without the use of any adhesives, non-woven tissue adheres well to concrete specimens.
- The workability and homogeneity of concrete were unaffected when non-woven tissues were applied as a layer on the exterior sides of concrete examples. However, the workability decreased very significantly as a result of the addition of cut pieces into concrete mixtures.
- The cube compressive strength is little affected by the attachment of non-woven tissue; however, the compressive strength of cylindrical samples strengthened with the tissue increased significantly. For the specimens having cut pieces, the compressive strength decreased significantly or high significantly. Additionally, unlike the reference samples, specimens with non-woven tissue did not break up into numerous pieces at the ultimate compressive force.
- Concrete beams reinforced with 1-layer, 2-sides, and 3-sides of non-woven tissue had greater flexural strength than the control beams. Additionally, control beams failed brittle and were split into two pieces, whereas non-woven tissue-covered beams displayed relatively ductile behavior, were not split into two parts, and had less widen cracks than control beams.
- At all curing regimes, the splitting tensile strength greatly improved for the cylinders covered with non-woven tissue compared to the reference cylinders. After the peak load, covered samples were maintained by non-woven tissue, while non-covered samples were separated into two distinct sections.
- Incorporation of non-woven cut pieces into concrete mixtures resulted in a remarkable decrease of dry density due to the lower density of non-woven fabrics and an increase of water absorption and porosity because of the weak transition zone, macro cracks, and voids present in the interfacial transition zone between plastic tissue and cement paste.
- The UPV findings fluctuated between 3.42 km/s and 4.64 km/s in the present study. The control specimens had the highest UPV value, whereas samples with non-woven tissue on 2-sides had the lowest UPV value. The addition of cut pieces greatly reduced the UPV value, and this reduction was more pronounced for higher percentages.
- The specimens containing layer or cut pieces of non-woven sheets had better thermal insulation than control specimens.
- Lastly, the relationships between various concrete properties were plotted and the R^2^ coefficient and line trend clearly show the great accuracy and indicated that certain properties of concrete could be predicted based on previously observed behaviors. Additionally, an earlier developed model was used to validate the new research data, which demonstrates excellent accuracy.
- Finally, this research study demonstrates that non-woven tissue can be employed in concrete since it adheres well to specimens and can enhance several concrete properties. Therefore, more research should be done to understand additional characteristics such as resistance in aggressive environments, microstructural analysis, durability, etc. However, additional research is also necessary to determine the mechanism behind the bond between non-woven tissue and concrete and especially the effect of water content, addition of superplasticizer, and type of surface on their bond properties. In addition, it is suggested to study the mechanical properties of concrete samples with two or more layers of non-woven sheets attached to one another using adhesive materials.

## Figures and Tables

**Table 1:** Ghori cement chemical composition.

Constituents	CaO	SiO_2_	Al_2_O_3_	Fe_2_O_3_	K_2_O	MgO	Na_2_O	S-	Cl-	SO_3_
**Weight (%)**	63.44	17.63	4.90	2.01	0.57	1.33	1.37	1.69	0.01	4.21

**Table 2:** Coarse and fine aggregates’ physical properties.

Type of Aggregates	Properties						
	Specific Gravity	Water Absorption (%)	Bulk Density (kg/m^3^)	Abrasion Value (%)			
**Fine aggregates**	2.653	1.051	1624.52	A	B	C	D
**Coarse aggregates (2–10) mm**	2.634	1.442	1288.90	23.97	29.86	28.86	27.16
**Coarse aggregates (6.3–25) mm**	2.586	1.128	1299.98				

**Table 3:** Physical and chemical properties of the ADIUM 150.

Properties	Value/Type
Color	Brown
State	Liquid
Density	1.03–1.07 kg/lit
pH	5.0 ± 0.5
Chloride content	Free
Alkali content	≤2.0% by weight

**Table 4:** Mix designs of concrete with and without cut pieces (kg/m^3^).

Materials	Weight of Materials for Different Percentage of Non-Woven Fabrics (kg)			
	0.0%	0.25%	0.50%	0.75%
Cement	455	455	455	455
Water	205	205	205	205
Sand	782	782	782	782
Coarse aggregate (10 mm)	358	358	358	358
Coarse aggregate (25 mm)	538	538	538	538
Non-woven sheets	0	5.33	10.66	15.99
Superplasticizer	2.5	3.6	5.0	6.1

**Table 5:** Type of samples and exposed tests.

Type of Samples and Their Dimensions	Using Method of Non-Woven Sheets	Name	Description	Conducted Tests	Image
Cube (100 mm)	Control specimen	Ref	No sheet	Compressive strength	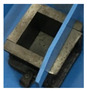
	As a layer	1-layer	A layer of non-woven tissue at the bottom		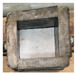
		2-sides	Non-woven tissue applied at two vertical opposite sides		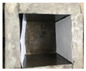
		4-sides (Full wrapped)	Non-woven tissue applied at all four sides		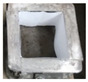
	As cut pieces	0.25%	0.25% of cut pieces of non-woven tissue		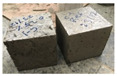
		0.50%	0.5% of cut pieces of non-woven tissue		
		0.75%	0.75% of cut pieces of non-woven tissue		
Cylinder (100 × 200) mm	Control specimen	Ref	No sheet	Compressive and split tensile strengths	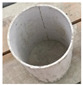
	As a layer	Layer	Entirely strengthened with non-woven tissue		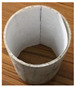
	As cut pieces	0.25%	0.25% of cut pieces of non-woven fabric		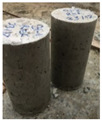
		0.50%	0.5% of cut pieces of non-woven fabric		
		0.75%	0.75% of cut pieces of non-woven fabric		
Beam (100 × 100 × 500) mm	Control specimen	Ref	No sheet	Flexural strength	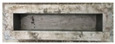
	As a layer	1-layer	A layer of tissue at the bottom		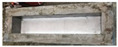
		2-sides	Tissue applied at two vertical opposite sides		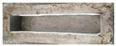
		3-sides	Tissue applied at two vertical opposite sides and bottom		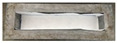
Cylinder (100 × 70) mm	Control specimen	Ref	No sheet	Dry density, porosity, water absorption, UPV, and thermal conductivity	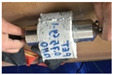
	As a layer	1-layer	A layer of tissue at the bottom		
		2-sides	Tissue at upper and lower sides		
	As cut pieces	0.25%	0.25% of cut pieces of non-woven fabric		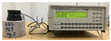
		0.50%	0.5% of cut pieces of non-woven fabric		
		0.75%	0.75% of cut pieces of non-woven fabric		

**Table 6:** The significance criteria for various properties of concrete.

Improvement or Reduction %	Significance Criteria
0–4.45%	Negligible
4.46–9.85%	Insignificant
9.86–25%	Significant
>25%	Highly significant

## Data Availability

The data presented in this study are available on request from the corresponding author.
